# A shared ‘vulnerability code’ underpins varying sources of DNA damage throughout paternal germline transmission in mouse

**DOI:** 10.1093/nar/gkad089

**Published:** 2023-02-20

**Authors:** Frances Burden, Peter J I Ellis, Marta Farré

**Affiliations:** School of Bioscience, University of Kent, UK; School of Bioscience, University of Kent, UK; School of Bioscience, University of Kent, UK

## Abstract

During mammalian spermatogenesis, the paternal genome is extensively remodelled via replacement of histones with protamines forming the highly compact mature sperm nucleus. Compaction occurs in post-meiotic spermatids and is accompanied by extensive double strand break (DSB) formation. We investigate the epigenomic and genomic context of mouse spermatid DSBs, identifying primary sequence motifs, secondary DNA structures and chromatin contexts associated with this damage. Consistent with previously published results we find spermatid DSBs positively associated with short tandem repeats and LINE elements. We further show spermatid DSBs preferentially occur in association with (CA)_*n*_, (NA)_*n*_ and (RY)_*n*_ repeats, in predicted Z-DNA, are not associated with G-quadruplexes, are preferentially found in regions of low histone mark coverage and engage the remodelling/NHEJ factor BRD4. Locations incurring DSBs in spermatids also show distinct epigenetic profiles throughout later developmental stages: regions retaining histones in mature sperm, regions susceptible to oxidative damage in mature sperm, and fragile two-cell like embryonic stem cell regions bound by ZSCAN4 all co-localise with spermatid DSBs and with each other. Our results point to a common ‘vulnerability code’ unifying several types of DNA damage occurring on the paternal genome during reproduction, potentially underpinned by torsional changes during sperm chromatin remodelling.

## INTRODUCTION

In mammals the production of fertilisation-competent sperm involves a drastic reduction in nuclear size—over 75% reduction in cell volume (1), to produce streamlined and hydrodynamic cells capable of fast independent motility. This dramatic loss of cytoplasmic content is accompanied by an equally drastic transformation of chromatin structure and organization (2). During the elongating stage of spermiogenesis, the genome is remodelled via the replacement of histones by protamines, resulting in a highly compact sperm nucleus and consequently the sperm head. While not all histone proteins are replaced—estimates for retention range from 1% to 10% in different species, fully protaminated chromatin packing is extremely space-efficient and approaches the theoretical crystal limit for DNA condensation (3).

Protamine bound DNA is less supercoiled than histone bound DNA because wider supercoils are produced (4,5). Consequently, the remodelling process requires significant changes in the DNA winding number to eliminate the free negative supercoils produced during histone replacement. This is believed to be enzymatically mediated by topoisomerase II beta (TOP2B) (or similar enzymes (6–8).These enzymes catalyse the scission of DNA strands to allow free rotation of the helix and/or unknotting of tangled strands. It is estimated that this chromatin remodelling requires between 5 and 10 million transient double strand breaks (DSBs) per cell (6). If these transient breaks are not correctly re-ligated by the enzyme generating them, this leads to damage that must be repaired by one of several DNA DSB repair processes (9,10).

Since round spermatids are haploid cells, DSBs cannot be repaired by homology-based mechanisms. Instead, breaks must be repaired by error prone mechanisms such as non-homologous end joining (NHEJ) or microhomology-mediated end joining (MMEJ) (11,12). NHEJ can result in errors when the free ends are ligated, leading either to small insertions or deletions or to wider genomic rearrangements if two distant DSBs are erroneously re-joined together. We have recently shown that spermatid DSB locations obtained by DNA Break Immunocapture (DBrIC, see Materials and Methods) are strongly correlated with evolutionary breakpoint regions (EBRs) (13) suggesting that a high proportion of all evolutionary genomic rearrangements may occur during spermiogenesis.

Any unrepaired breaks remaining in the mature sperm have the potential to affect the next generation, if not repaired by the oocyte. For example, increased sperm DNA fragmentation index (DFI) is associated with miscarriage (14). As such, determining the context in which DSBs occur within mouse spermatids (post meiotic cells) is a pivotal question both for understanding evolutionary transformations in genome structure and also for delineating the vulnerability of different regions of the sperm genome to clinically significant DNA damage.

Previous work in this field (15) has shown that spermatid DSBs are not randomly distributed, but rather are associated with particular categories of genomic repeats (LINE, satellite and simple repeats). Further work looking at mature sperm has shown that genomic repeats, in particular SINEs, are also enriched for oxidative damage occurring during epididymal maturation, and that this form of damage is also correlated with histone retention in sperm, and with 3D localisation in the sperm nucleus (16). It is as yet unclear whether there is a common ‘vulnerability code’ in which particular sequence or topological features underlie the susceptibility of sperm chromatin to multiple different types of damage at successive developmental stages.

In this study, we aim to determine whether mouse spermatid DBrIC DSB locations are unique to this cell type or found in common with other cell types, to investigate whether common or cell type specific breaks occur within similar or different sequence contexts, to extend the previous analysis of DSB distribution to a fuller consideration of chromatin structure and DNA topology in the male germline, and to determine whether chromatin at risk of DSBs during sperm head compaction is also prone to oxidative damage in the mature sperm head.

## MATERIALS AND METHODS

### DSB detection by DBrIC (spermatids)

To study spermatid DSB locations, DSB data obtained by DNA break Immunocapture (DBrIC) was analysed as previously described (13). DBrIC involves nick and gap repair using T4 ligase and polymerase. The DSBs that remain are labelled with TdT and biotin-14-dATP. The DNA is fragmented using Shearase and immunoprecipitated. This technique does not show precise DSB locations, but indicates the near vicinity of each labelled DSB, with the resolution governed by the size of the sheared DNA fragments. Three fastq files were downloaded from NCBI ERR1886418 and ERR1886419 (spermatids steps 1–9), and ERR1886420 (spermatids steps 15–16) (15) (Supplementary Table S1). Read quality was checked using FastQC (v0.11.9, Andrews 2020). Reads were trimmed using Trimmomatic (v0.39) (17) with the SE setting. Adapter sequences were removed using ILLUMINACLIP, as well as reads with an average Phred score of < 30 with the AVGQUAL:30 or reads with average length < 30 using MINLEN:30. Trimmed fastq files were aligned to the mouse genome mm10 using bwa-mem with default parameters (v0.7.17-r1188, Li, 2013).

**Figure 1.**
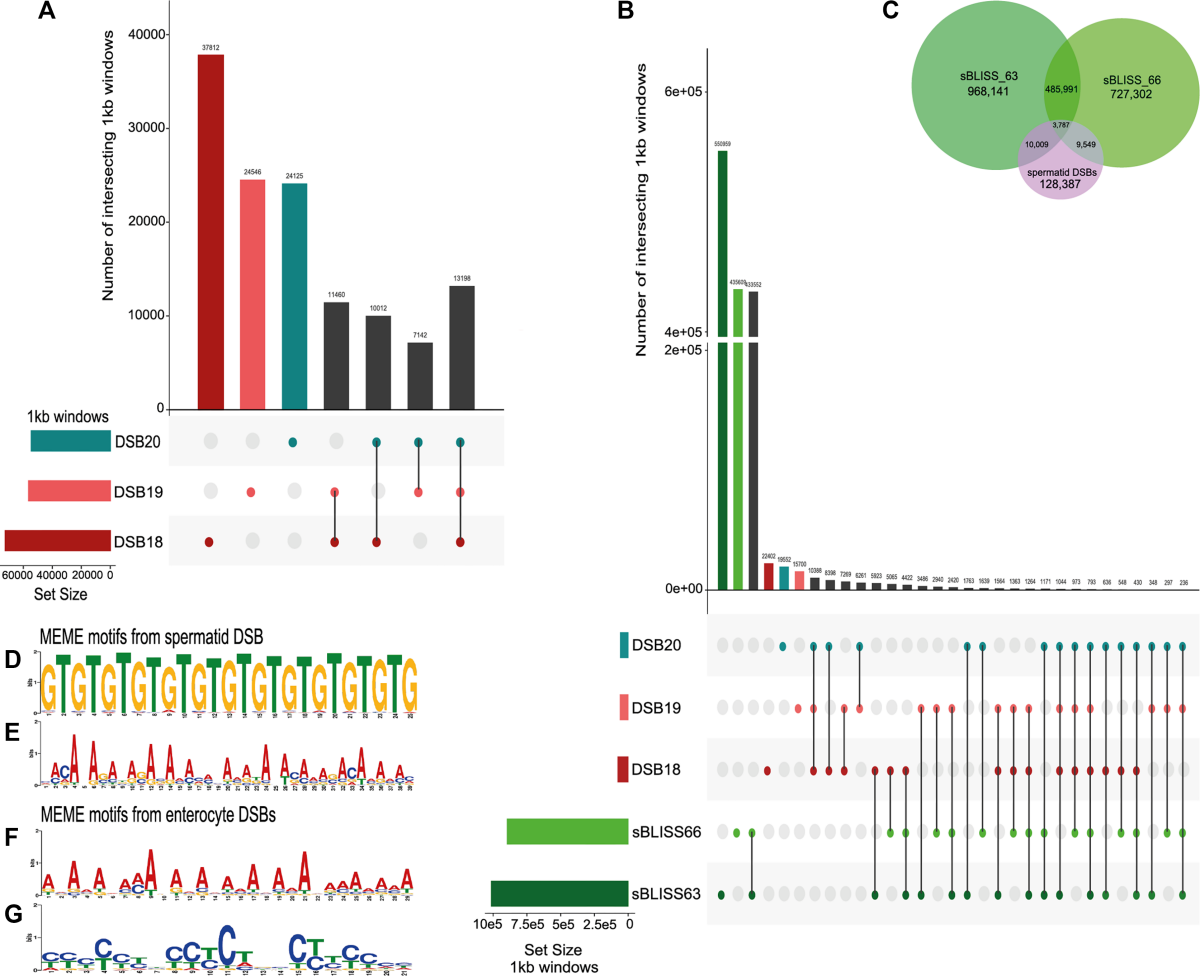
(**A**) A 1 kb UpSetR plot showing the overlap of the three spermatid DSB files merged to create the spermatid DSB locations track. The number of 1 kb windows with a DSB signal in each file is represented on the left barplot as ‘set size’. The X-axis represents the number of 1 kb windows containing a DSB signal for the different overlap combinations. Different combinations of overlap are represented by the black lines interlinking the coloured circles. The DSB18/19 files are round spermatids stages 1–9 (shown in red and pink bars) and represent the total number of 1 kb windows containing a signal unique to these files. The DSB20 file (shown in the blue bar) is condensing spermatids stage 15–16 and this peak also represents the total number of 1 kb windows containing a DSB unique to this file. Bars in dark grey represent the number of 1 kb windows with signal in more than one file. (**B**) A 1 kb UpSetR plot showing the overlap of the three spermatid DSB files 18/19/20 (with the same colour shading as shown in (A)) and the enterocyte sBLISS63/sBLISS66 files shown in green. The two green bars represent the total number of 1 kb windows containing a sBLISS63 or sBLISS66 peak unique to these files. The enterocyte files used were the 1 bp DSBs as per the original bed files. (**C**) Venn diagram of the spermatid DSB locations file and the extended sBLISS_63/66 files (extended to the mean size of the spermatid DSB locations). Not to scale. (**D** and **E**) Spermatid DSB MEME motifs, ordered by decreasing E-value. The input file to MEME contained all spermatid DSB locations not just the spermatid specific ones. (**F** and **G**) The two most common motifs from MEME for all the extended enterocyte DSBs, ordered by decreasing *E*-value.

Peaks were called using the same parameters described in the original study (15). Briefly, MACS2 with the default settings and *–bw600 -q0.01 –broad –broad-cutoff 0.1* were used (see Supplementary Table S2 for peak statistics). We then examined the overlap of the peaks of the different DSB files. A 1kb windowed UpSetR analysis showed a good correlation between the different DSB files (Figure 1A). A 5kb windowed UpSetR analysis showed more overlap, as expected (Supplementary Figure S1). A 1kb corrplot (Supplementary Figure S2) also showed a good correlation. Therefore, MACS2 files of the different round spermatid stages (stage 1–9 and stage 15–16) were merged for further analysis to facilitate cross-comparison with datasets not stratified into different developmental stages in spermatids. The longest DSBs peaks were visualised in IVG and co-located with large stretches of alpha satellite regions. Because alpha satellites are not fully assembled in the centromeres of all mouse chromosomes, to avoid bias towards the assembled stretches of satellite regions, we excluded these DSBs peaks from further analysis.

### DSB detection by sBLISS (enterocytes) and comparison to spermatid DBrIC data

To study enterocyte DSB locations, sBLISS (in-Suspension Break Labelling In Situ and Sequencing) data was downloaded from NCBI (GSE145598) (18) and used as an alternative source of DSB data to compare to the spermatid DBrIC DSB locations data. In sBLISS, the cells are harvested, fixed and crosslinked. DSB ends are then blunted and adapters are ligated, followed by next generation library preparation and sequencing. This method does not require an immunoprecipitation step. sBLISS also indicates the precise location of each detected DSB. Both sBLISS files used were replicates from enterocytes with high levels of Cd73/Nt5e, representing cells from the tip of the villus. For an initial comparison of the degree of concordance between spermatid DBrIC DSB locations and enterocyte DSB locations, the enterocyte files (GSM4322063 termed sBLISS 63 and GSM4322066 termed sBLISS 66) were analysed separately and the DSB locations analysed at maximum (1bp) resolution (Figure 1B). For further analysis both the sBLISS 63 and sBLISS 66 files were extended 133 bp upstream and 133 bp downstream to simulate peaks the same size as the mean of the peaks obtained in the spermatid DBrIC data. Any peaks overlapping regions of centromeric satellite repeats (GSAT_MM from the RepeatMasker track) were removed. *Bedtools intersect* (version bedtools2-2.29.2) (19) was used to obtain a unique file of the extended sBLISS 63 peaks that overlapped the extended sBLISS 66 peaks. This file was then used for motif analysis and permutation testing. We finally used this file with bedtools intersect to identify spermatid DSB peaks not overlapping enterocyte DSBs.

### DSB motif analysis

MEME 5.1.1 (20), was used to search for the top three motifs in the merged spermatid DSB sample (Figure 1D, E) with the option *–revcomp*, using both the given strand and the reverse complement strand when searching for motifs. The same settings were used to search for motifs in the spermatid specific breaks, enterocyte specific breaks, shared spermatid/enterocyte breaks and all enterocyte breaks (Supplementary Table S3).

*Bedtools* *getfasta* was used to extract the sequence contained within the extended enterocyte DSB peak file described above (Figure 1F, G).

### Oxidative damage data

Paired end cauda epididymal sperm oxidative damage (OD) data was obtained from PRJEB11644 (16). Data from file ERR1162982 with a gpx5 mutation was termed moderate OD and data from file ERR1162983 with both a gpx5 and sngpx4 mutation was termed severe OD. A windowed data analysis approach was used to calculate the amount of oxidative damage in 50 kb windows (21). Briefly read 1 and read 2 fastq files were independently aligned to the mouse genome mm10 using bwa-mem (v0.7.17-r1188). The bam files were sorted and read 1 read 2 files were merged using *samtools merge* (22) to treat the files as SE as per the original paper. 50kb windows of the mm10 genome were created using *bedtools* *makewindows* (19). *Bedtools coverage* (19) with the *–mean* was used to calculate the mean coverage at each 50 kb window. A global scaling factor was calculated as the mean read coverage in a low-damage subset (10%) of the 50 kb bins, these bins had the lowest 10% read coverage in the 50 kb windows over the mean of all three files downloaded. This global scaling factor was used in a custom Perl script to calculate scaled OD values per 50 kb window of the mm10 genome. Relative enrichment of DNA damage was assessed through the fold change of the enriched OD samples over input WT sample.

Any 50 kb window of the genome which overlapped with GSAT_MM regions from the RepeatMasker track was removed from both OD files. The top 1% of the highest damaged windows were used for further analysis (Supplementary Table S4).

### Non-B DNA sequences

Predicted Non-B DNA sequences (Z-DNA, STR and G-quadruplex) were downloaded from the Non-B DNA Database (https://nonb-abcc.ncifcrf.gov/apps/Query-GFF/feature/) from the mm10 genome. Data for each chromosome was merged into a master file. For the predicted Z-DNA and STR samples *bedtools intersect* (19) was used with the –v option to obtain a file of predicted Z-DNA regions that did not overlap STR and a file of STR regions that did not overlap predicted Z-DNA. While experimentally validated G-quadruplex regions were obtained from GSE110582 (23).

### Histone marks

Fastq files from either round spermatids or epididymal sperm (24–28) were downloaded from NCBI (Supplementary Table S1). Read quality was checked using FastQC (v0.11.9, Andrews, 2020). Reads were trimmed using Trimmomatic (v0.39) (17) with the following settings (SE, ILLUMINACLIP and AVGQUAL:20). Trimmed fastq files were aligned to the mouse genome mm10 using bwa-mem (v0.7.17-r1188, Li, 2013).

Histone mark data was analysed using MACS2 (v.2.2.7.1) (29) with either the default settings of *callpeak* to produce narrow peaks or with *–broad –broad-cut-off 0.05* to produce broad peaks. Histone marks were defined as narrow or broad based on the ENCODE project (30). For marks not defined on ENCODE, the broad settings were used (see Supplementary Table S2 for peak statistics and Supplementary Figure S3 and Supplementary Table S5 for coverage of histone marks per chromosome).

### ChromHMM

ChromHMM (v1.22) (31) was used for chromatin state analysis of the spermatid ChIP-seq data. The default binsize of 200 bp was used with the concatenated strategy. The corresponding cell type specific input was used as a control to adjust the binarization threshold locally. Once binarization was completed the model was learned with varying numbers of states, with 16 states chosen as the optimum model (Figure 3). See Supplementary Table S7 and Supplementary Figure S4 for coverage of the states per chromosome.

### Permutation tests

RegioneR (1.22.0) (32) run with R 4.0.3 was used for permutation testing between the merged spermatid DBrIC DSB locations file and the histone ChIP-seq data or files of DNA structure (Supplementary Table S6). Randomization was carried out per standard chromosome with an unmasked mm10 genome, 1000 permutations and *nonoverlapping = FALSE*.

Genomic association tester (GAT 1.3.4) (33) was used to compute the fold change between the merged spermatid DBrIC DSB locations file, the top 1% of moderate and severe OD damage regions, the histone ChIP-seq data and files of DNA structure (Supplementary Figure S5). The spermatid DBrIC DSB locations file, OD damage, non-B DNA files or histone ChIP-seq files were used as the segment file, the workspace was the mm10 genome and the annotation file was either the DBrIC DSB locations file, OD damage, a histone ChIP-seq file, or a file of DNA structure. All iterations were run with *–num-samples = 10000*.

### Data visualization

Circular plots were created using the R tool circlize_0.4.15 (34) within R 3.6.1 with the outer track as the mm10 Ideogram. The UpSetR plots were created using the R tool UpSetR_1.4.0 (35) within R 3.6.1 using either 1kb or 5kb binarized DSB data as input. PyGenomeTracks-3.6 (36,37) was used to visualize the same genomic regions from different browser tracks, to illustrate regions of overlap between samples. The PyGenome scripts make_tracks_file and pyGenomeTrack were used. Heatmaps were generated in R 4.0.3 using the program pheatmap (v1.0.12, Kolde, 2019). The 1kb DSB corrplot was created using the R tool corrplot (v0.92, Wei and Simko 2021) within R 4.0.3.

## RESULTS

### Genomic distribution of spermatid DSBs

We used publicly available DSB data (Supplementary Table S1) from spermatids: these comprised two replicates of round spermatids at developmental steps 1–9 (DSB18 and 19), and one of condensing spermatids at developmental steps 15–16 (DSB20). We first tested the concordance between the three spermatid files by detecting overlaps in genomic windows of high resolution (1 kb, Figure 1A) and moderate resolution (5 kb, Supplementary Figure S1). This showed strong agreement between all three files. Around half the genomic windows containing a DBrIC signal in any given file also contained a DBrIC signal in at least one of the other files (47.8–56.4% overlap at 1 kb resolution, 55.2–68.6% overlap at 5 kb resolution). Importantly, the overlap between round and condensing spermatid data was as close as the overlap between the two round spermatid replicates, indicating that DSBs occur in similar genomic locations throughout different stages of spermatid development. We therefore combined the three DBrIC files in all subsequent analyses to yield a single set of spermatid DBrIC peaks.

**Figure 2.**
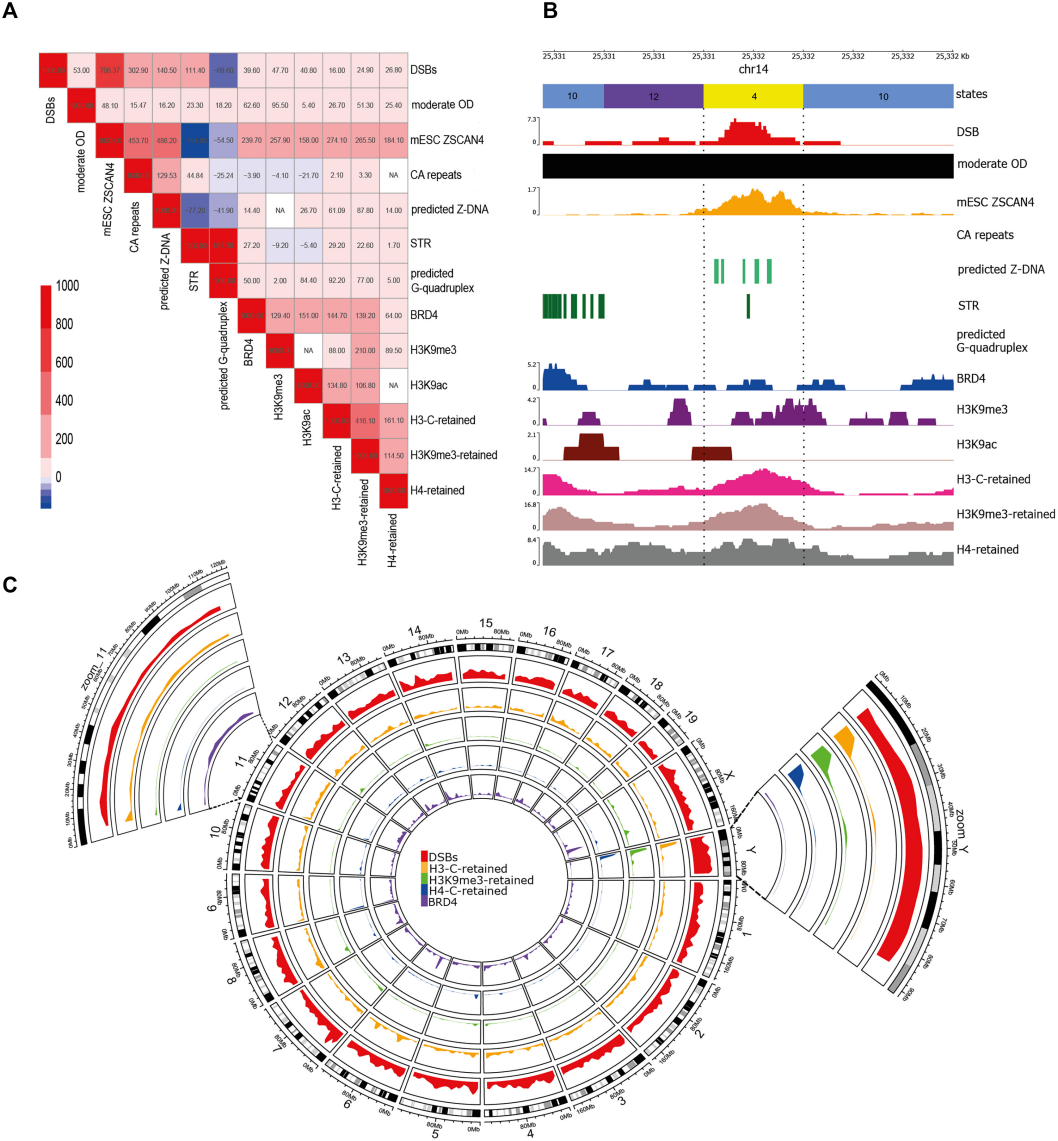
(**A**) Heatmap plotting the *Z*-score of spermatid DSB locations showing the association with different classes of non-B DNA. For plotting the associations in red on the diagonal have been fixed at 1000. Non-significant values are in white, and all coloured cells have a *P*-value of ≤0.05. Red shading represents a significant positive association and blue shading a significant negative association. The OD sample is the moderate OD damage, top 1% of 50 kb regions. (**B**) Example of a genomic region showing association of a spermatid DBrIC peak with ChromHMM states, oxidative damage, mESC ZSCAN4, CA repeats, predicted Z-DNA, STR, predicted G-quadruplex, BRD4, H3K9me3, H3K9ac and retained histones. (**C**) Circos plot showing the distribution of spermatid DSB locations across the genome (red track). The orange, green and blue tracks show the distribution of retained histones in sperm and the purple track shows the distribution of spermatid BRD4. The two extra panels are enlarged tracks for chr11, which has the lowest coverage of spermatid DSB locations and chrY with the highest spermatid DSB location coverage.

As previously described (13), from the combined files we identified a total of 151,732 post-meiotic DSB locations in spermatids, covering 1.49% of the mouse genome (Figures 1A and 2C). The DBrIC signal peaks ranged from 146 to 6662 bp, with a mean and a median of 267 and 213 bp, respectively. The coverage of DBrIC peaks per chromosome was scaled per Mb and plotted against chromosome length. This showed that chr11 and chrY were outliers with chromosome 11 having the lowest coverage of DBrIC peaks (0.97%) while the Y chromosome had the largest (3.51%) (Supplementary Figure S6). The mean number of DBrIC peaks per Mb genome wide was 56, whereas for chr11 it was 37 and for chrY it was 144. Consistent with published findings (15), the post meiotic DBrIC peaks were also associated with repeat content within each chromosome (*r*^2^ = 0.78, *P* = 0.00003) and were enriched for simple repeats and satellite regions (Supplementary Figure S7). Specifically, we found post-meiotic DBrIC peaks co-localise with transposable elements L1Md_T and L1Md_A (12.6 and 11.1 fold, *P* = 0.001). ChrY has the second highest coverage of RepeatMasker elements per Mb and chr11 has the second lowest. Given that spermatid DBrIC peaks are associated with repeat content this may partly explain why chrY has the highest coverage of spermatid DBrIC peaks and chr11 the lowest. For simplicity, we refer to spermatid DBrIC peaks hereafter as ‘DSB locations’, but note that this does not mean the breaks occur at precisely localised sites.

### Identifying spermatid specific DSBs

We classified spermatid DSB locations as uniquely found in spermatids (spermatid-specific) or shared with other cell types by overlapping spermatid DSB locations with two publicly available files (sBLISS 63 and sBLISS 66) covering DSB localisation in developing enterocytes, as detected via sBLISS (18) (Supplementary Table S1). It is important to note that the methodologies used to detect DSBs in each cell type differ: DBrIC is lower resolution and requires an immunoprecipitation step (see Methods) while sBLISS is higher resolution and utilises adapter ligation in situ. Neither allows direct quantitation of the absolute number of DSBs per cell, but both allow analysis of the distribution of DSB locations across the genome. With these caveats noted, we observe that DSBs in spermatids as measured by DBrIC show a more restricted distribution than enterocyte DSBs as measured by sBLISS, with a smaller number of locations present in the genome (Figure 1B). Intriguingly, only a small fraction, (3.7% for sBLISS 63 and 3.6% for sBLISS 66) of spermatid DSB locations overlapped with enterocyte sBLISS DSBs, while 96.3% and 96.4% represented spermatid-specific DSBs (Figure 1B). Conversely, 99.6% of both sBLISS 63/66 1bp DSBs did not overlap spermatid DSB locations. We considered whether this lack of overlap could be caused by the differing resolution of DBrIC versus sBLISS data. To adjust for this factor, we extended the enterocyte sBLISS data ±133 bp either side of the detected DSB sites to match the average length of the spermatid DBrIC peaks and recalculated the overlap (Figure 1C). This showed that only 9.1% and 8.8% of the spermatid DBrIC peaks overlapped the extended sBLISS 63 regions and the extended sBLISS 66 regions, respectively. We conclude that DSB locations in both cell types are overwhelmingly cell type specific, and thus that the processes leading to DNA damage in spermatids and enterocytes are likely to be largely distinct, but that a small number of genomic regions are liable to breakage in both cell types.

### Spermatid DSB locations occur in association with (CA)_*n*_ and (NA)_*n*_ motifs

Having defined DSB locations for each data set, we used MEME (20) to identify specific DNA sequence motifs associated with the presence of DSBs in each cell type (Supplementary Table S3). For the enterocyte dataset, we used the extended (±133 bp) sBLISS data, to ensure that we were comparing a similar-size genomic region for each DSB location detected in each cell type. This analysis therefore will detect DNA sequence motifs found in the near vicinity of DSBs for each cell type. Considering all spermatid DSB locations together, an alternating purine pyrimidine sequence ((CA)_*n*_ or equivalently (GT)_*n*_) and a more degenerate alternating (NA)_*n*_ motif (Figure 1D, E) were identified as being statistically over-represented relative to a random model adjusted for nucleotide frequency. The (CA)_*n*_ motif is present in 43.2% of the spermatid DSB location peaks, and the (NA)_*n*_ motif is present in 62.4% of the peaks. In contrast, considering all the enterocyte DSB locations together, neither (CA)_*n*_ nor (NA)_*n*_ sequences were detectably enriched relative to a random model adjusted for nucleotide frequency. Instead, an A-rich motif was detected in 42.9% of the enterocyte DSB locations and a C-rich motif in 38.4% (Figure 1F, G). This again suggests that there are likely to be distinct damage sources at play in each cell type.

We then refined this analysis by searching for motifs specifically within spermatid-specific, enterocyte-specific and shared DSB locations as defined above (Figure 1C, Supplementary Table S3). This confirmed the enrichment for (CA)_*n*_ and (NA)_*n*_ motifs in spermatid-specific DSB locations (40.6% and 32.4% of these locations containing each motif respectively). T-rich and C-rich motifs were also confirmed as enriched in enterocyte-specific DSB locations (30.6% and 25.7% of these locations containing each motif respectively). In this sub analysis, a (CA)_*n*_ motif was also detected as enriched in enterocyte-specific DSB locations, but to a much lesser degree, with only 5.3% of enterocyte-specific DSB peaks containing this motif. There was no enrichment for an (NA)_*n*_ motif in enterocyte-specific DSB locations. For shared DSB locations found in common across all cell types, the (CA)_*n*_ and (NA)_*n*_ motifs were very highly enriched (86.9% and 72.9% of locations respectively), but the T-rich and C-rich motifs were not detectably enriched. Overall, 17.2% of all (CA)_*n*_ repeats in the genome overlapped with spermatid DSB locations, while 15.1% and 12.8% overlapped with the extended enterocyte sBLISS 63/66 DSBs. Thus, (CA)_*n*_ repeats appear to be a common fragility motif in both cell types studied, but slightly more so in spermatids than in enterocytes.

Alternating purine-pyrimidine repeats have previously been described as topoisomerase II cleavage sites (38). We therefore tested whether there was a general association between motifs with alternating purine/pyrimidine sequences (positive strand only) and spermatid post-meiotic DSB locations (Supplementary Table S6). Using a 9-repeat motif length, (RY)_9_, there was a significant positive association (*Z*-score = 29.1, *P* = 0.001, 1000 permutations). When extending the RY motif to 26 repeat units, (RY)_26_, the mean length of the (CA) repeats in the mouse genome, the association was more significant (*Z*-score 35.8, *P* = 0.001, 1000 permutations). The positive association remained when excluding any (RY)_26_ repeats that overlapped any (CA) repeat in the genome (*Z*-score 33.1 *P* = 0.001, 1000 permutations). Therefore, we can conclude that in spermatids, DSB locations are associated with (RY) repeats, in particular those with alternating A residues (or equivalently alternating T residues), and most strongly in the context of (CA)_n_ repeats. However, using permutation testing, we observed a negative association (*Z*-score = –24.4, *P* = 0.001, 1000 permutations) between the topoisomerase II consensus motif (RNYNNCNNGYNGKTNYNY) and spermatid post-meiotic DSB locations. Thus, the association with RY repeats is not driven by the canonical topoisomerase II consensus motif.

### Spermatid DSB locations and enterocyte DSBs are associated with distinct topological configurations of DNA

Our initial analysis (Figure 1D–G and Supplementary Table S3) revealed different classes of simple repeat motifs associated with DSBs in each cell type. These repetitive motifs, such as short tandem repeats (STRs) are more likely to fold into non-canonical DNA structures. While DNA in cells typically folds into the widely known B-form with a right-handed helical structure, it is known that (CA)_*n*_ sequences can readily undergo a transition to a left-handed Z-DNA conformation when subjected to unwinding torsional stress (39,40). Therefore, we determined whether each of our DSB categories was associated with STRs or with regions predicted to fold into Z-DNA (Table 1 and Supplementary Table S6).

**Table 1.** Correlation between different classes of non-B DNA and spermatid or enterocyte DSBs. Values reported are *Z*-scores using 1000 permutations and *P*-value <0.001

Type	All spermatid DSB locations	Spermatid specific DSB locations	Shared DSBs	Enterocyte specific DSBs	All enterocyte DSBs
Predicted Z-DNA No overlap STR	150.7	125.6	61.2	63.8	65.2
STR No overlap predicted Z-DNA	110.9	104.8	42.1	–25.2	–23.8
Predicted Z-DNA overlapping STR	1367.7	1303.9	533.2	–22.9	–5.7
Predicted G-quadruplex	–72.9	–68.3	–12.5	21.5	19.0
G-quadruplex experimental	–32.5	–35.1	5.6	108.6	109.9

* All *P*-values were 0.001.

To disentangle the effect of primary DNA sequence from that of DNA secondary structure, we tested separately for associations between each category of DSB and STRs predicted to form Z-DNA, STRs that are not predicted to form Z-DNA, and non-repetitive regions that are predicted to form Z-DNA. We also tested for any association with G-quadruplexes, as these have also previously been associated with DNA damage (10).

Spermatid-specific DSB locations were strongly and independently positively associated with STRs and with predicted regions of Z-DNA, but negatively associated with G-quadruplex forming regions. Enterocyte-specific breaks were positively associated with experimentally determined G-quadruplexes, computationally predicted G-quadruplexes and non-repetitive predicted Z-DNA regions, but negatively associated with STRs and with predicted repetitive Z-DNA forming regions. Shared breaks were positively associated with all features except computationally predicted G-quadruplexes.

Given that the spermatid-specific and shared spermatid-enterocyte DSB locations exhibited the same primary sequence motifs, with very similar associations with predicted secondary structures, we therefore pooled these together for subsequent analysis of their epigenetic chromatin context. Enterocyte-specific breaks were not addressed further in this study.

### Spermatid DSB locations co-locate with markers of NHEJ but are negatively associated with most other histone modifications

Having investigated the primary and secondary DNA sequence features associated with DSBs, we turned our attention to whether the epigenetic landscape affects the location of DSBs. Using publicly available data for 16 different epigenetic marks in round spermatids (Supplementary Table S1), H3K4me1 showed the highest coverage genome wide (5.9%) while Kac had the lowest (0.001%) (Supplementary Table S5 and Supplementary Figure S3). Only BRD4, H3K9me3, H3K9ac, Kac and H2AZ were positively associated with spermatid DSB locations (with *Z*-scores of 39.6, 47.7, 40.8, 4.9 and 18.0, *P* = 0.001 with 1000 permutations), while H4K5ac, H4K8ac, H4K12ac, H4Kac, H3K4me3, H3K27me3, Kcr, H3K27ac, H3K4me1 and 5hmC were negatively associated and H4K16ac was not significantly associated (Figure 2, Supplementary Figure S5 and Supplementary Table S6).

**Figure 3.**
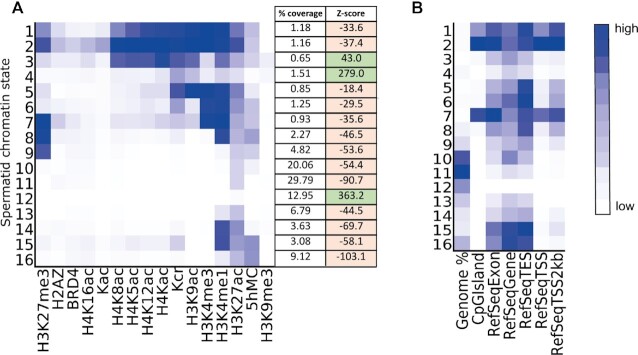
(**A**) ChromHMM emission plot showing spermatid chromatin states E1–E16 with genome coverage (%) and *Z*-score results of permutation tests between spermatid post-meiotic DSB locations and ChromHMM states. In green are ChromHMM states with a significant positive association with spermatid DSB locations and in pink are states with a significant negative association with spermatid DSB locations. All *Z*-score *P*-values were 0.001. (**B**) ChromHMM overlap plot showing fold enrichment with various refseq categories.

To assess whether spermatid DSB locations occur in a specific chromatin context, we first ran ChromHMM (31) with 16 histone marks. At 200 bp resolution, a total of 16 different chromatin states were identified (Figure 3A). The three states with the highest genomic coverage were 10, 11, 12 (20.06, 29.79 and 12.95% coverage) Figure 3B. State E12 was notable for low coverage of all histone marks, while state E2 had the highest coverage of all marks. Coverage of all states varied in each chromosome, with state E12 having the lowest coverage on chr 11 (8.65%) and the highest on chr 3 (15.02%). State E3 had the lowest coverage on chrY (0.2%) and the highest coverage on chr17 (0.83%), while state E4 had the lowest coverage on chr 11 (1.16%) and the highest on chrY (2.19%) (Supplementary Table S7 and Supplementary Figure S4).

Only states E3, E4 and E12 were positively associated with spermatid DSB locations (Z-scores 43.0, 279.0, 363.2, *P* = 0.001, 1000 permutations) (Figure 3A and Supplementary Table S6). Our results thus far suggest that spermatid DSB locations are strongly associated with the (CA)_*n*_ motif, tend to occur in genomic regions with low coverage of histone modifications (state E12, Figure 3) and are located in regions where BRD4 is found. A positive association between BRD4 and H3K9me3 (both part of the DNA damage response), and spermatid DSB locations provides further supporting evidence that the DSB locations we identify are indeed related to DNA damage and not spurious associations. Notably BRD4 is specifically associated with NHEJ (41) rather than other DSB repair pathways, consistent with the lack of homologous recombination and the requirement for NHEJ in spermatid DNA repair.

### Spermatid DSB locations are associated with histone retention and oxidative damage in mature sperm

The presence of DSBs in spermatids may affect subsequent downstream events in spermatogenesis either directly (if DSB formation and repair interferes with protamination) or indirectly (if both DSB formation and protamination are affected by the same underlying genomic features). Therefore, we investigated the association of spermatid DSB locations with regions retaining histones in mature sperm (Figure 2). Our results showed that post-meiotic DSB locations are enriched in retained histones in mature sperm (*Z*-scores of 16.0, 24.9 and 26.8, *P* = 0.001, 1000 permutations for H3-C, H3K9me3 and H4 replicate 2 respectively). As such, DSB locations may have a lower rate of histone to protamine replacement or the inability to undergo repackaging.

We also investigated the correlation of predicted Z-DNA with regions retaining histones in mature sperm. H3-C, H3K4me3 and H3K9me3 replicate 2 are all positively associated with *Z*-scores of 61.1, 81.6, 87.8 (*P* = 0.001, 1000 permutations) (Supplementary Table S6).

As has been previously described, reactive oxygen species (ROS) such as hydrogen peroxide are required for further chromatin compaction as they are essential for the formation of protamine-to-protamine disulfide bond formation (42). However, altered DNA packaging in round spermatids or sperm may increase its vulnerability to oxidative damage during epididymal maturation. Therefore, using publicly available data from two mouse genotypes with moderately and severely increased susceptibility to oxidative damage (OD) in mature sperm (Supplementary Table S1) (16), we determined whether regions of high OD co-localise with spermatid DSB locations—i.e. whether pre-existing damage in spermatids may precondition mature sperm for further damage. Oxidative damage in sperm is in general not concentrated into tight hotspots but occurs across broader regions reflecting larger scale variations in packaging properties. Therefore, following previous publications (21), we divided the mouse genome into 50 kb windows and identified the top 1% of windows with the highest oxidative damage for both the moderate and severely damaged samples (see Materials and Methods). Chromosome 5 had the highest coverage for both samples while chromosome Y the lowest (Supplementary Table S4).

Overall, moderate and severe OD regions correlate with spermatid DSB locations (*Z*-score = 27.1 and 27.4, *P* = 0.001, 1000 permutations), as well as predicted Z-DNA and STRs (with *Z*-scores of 16.2 and 23.3, *P* = 0.001, 1000 permutations for moderate OD respectively) (Supplementary Table S6). However, OD regions also showed a positive correlation with predicted G-quadruplexes that was not observed for spermatid DSB locations (*Z*-score = 18.2). To assess whether the associations of OD regions with non-B DNA were related to the co-localization of non-B DNA regions with spermatid DSB locations, we removed any non-B DNA regions that overlapped the spermatid DSB locations and repeated the analysis. Both moderate and severe OD regions still gave positive correlations with predicted Z-DNA, STR and G-quadruplexes (with *Z*-scores of 10.7, 16.9 and 17.6, *P* = 0.001, 1000 permutations for moderate OD) (Supplementary Table S6).

### The transcription factor ZSCAN4 in embryonic stem cells with two-cell like features is positively associated with spermatid DSB locations and regions of high oxidative damage in sperm

In the post-fertilisation embryo, protamine packaging of the paternal DNA must be replaced by histone proteins. This may once again make the genome vulnerable to DNA damage from torsional changes and/or topoisomerase activity. ZSCAN4 is a transcription factor which occupies a subset of (CA)_*n*_ microsatellite repeats in their nucleosomal form in mouse two-cell embryos (43), and is thought to help protect these fragile regions from genomic instability. Therefore, we investigated whether ZSCAN4 bound regions in E14Tg2a (E14) mouse embryonic stem cells (mESCs) were associated with spermatid DSB locations and with sperm oxidative damage.

Two-cell like ZSCAN4 regions in the mouse genome were not uniformly distributed, with chromosome 11 having the highest coverage of two-cell like ZSCAN4 (36.2%) and chromosome Y the lowest (13.5%) (Supplementary Table S8). Our results indicate a very large positive association between post-meiotic spermatid DSB locations and embryonic stem cell two-cell like ZSCAN4 (*Z*-score = 706.4, *P* = 0.001, 1000 permutations, Figure 2A and Supplementary Figure S5). Because ZSCAN4 has been shown to be associated with (CA)_*n*_ repeats, we tested whether our association is driven solely by these repeats, or whether there is an independent association between spermatid DSB locations and ZSCAN4 binding in ESCs. To do so, we then separated spermatid DSB locations that overlap CA repeats then those that do not overlap and re-ran permutation testing. Both analyses showed a positive association, with the significance of the association being higher for spermatid DSB locations that do not overlap CA repeats (*Z*-score = 629.2, *P* = 0.001, 1000 permutations) than for spermatid DSB locations that overlap CA repeats (*Z*-score = 389.6, *P* = 0.001, 1000 permutations). Since the association appeared not to depend on the primary sequence motif, we therefore also looked at the association of predicted Z-DNA forming regions to two-cell like ZSCAN4. Predicted Z-DNA regions that overlapped spermatid DSB locations were strongly positively associated with two-cell like ZSCAN4 (*Z*-score = 356.2, *P* = 0.001, 1000 permutations). Furthermore, the top 1% of 50 kb moderate OD damaged regions were positively associated with two-cell like ZSCAN4 (*Z*-score = 48.1, *P* = 0.001, 1000 permutations) (Supplementary Table S6).

Mouse embryonic stem cell two-cell like ZSCAN4 regions and regions retaining H3K9me3 in mature sperm are strongly positively associated (*Z*-score 1131.0 *P* = 0.001, 1000 permutations). This may suggest that regions retaining histones represent fragile genomic regions in a two-cell embryo.

## DISCUSSION

There is a widely known paternal mutation bias, widespread across amniotes and initially ascribed to the higher number of cell divisions in the paternal line, but which has recently been shown to be partly independent of cell division (44). Paternal specific events during post-replicative stages of gametogenesis and fertilisation are attractive candidate mechanisms for a cell division independent contribution to this paternal mutation bias. These include the extensive chromatin remodelling occurring during paternal genome condensation and decondensation, and also the specific exposure of the paternal genome to oxidative damage during epididymal transit and fertilisation. This paternal mutation bias is mirrored by an increasing appreciation of the role of sperm DNA fragmentation as a cause of male sterility, in particular as a cause of recurrent miscarriage and IVF failure (14,45). It is therefore important to understand the factors underpinning DNA damage occurrence and localisation in the male germ line, as any damage remaining in mature sperm that cannot be repaired by the oocyte has the potential to affect a developing embryo, with both clinical and evolutionary consequences.

Here, we expand on previous investigations exploring the distribution of spermatid post-meiotic DSB locations. Our investigation first compared spermatid and enterocyte DSBs to establish the tissue-specific nature of spermatid DSB locations and investigated both primary sequence motifs and secondary DNA structure associations in each cell type. This showed that the patterns of breakage are cell type specific, and in particular that spermatid DSB locations are associated with (CA)_*n*_/(NA)_*n*_ repeats and predicted Z-DNA, while enterocyte DSBs are associated with poly-T, G-rich regions and G-quadruplexes. The small proportion of shared breaks showed a similar motif pattern to spermatid specific breaks. We conclude that the association with predicted Z-DNA and simple repeats is driven by a process that is very prominent in spermatids but less so in enterocytes—most likely the huge torsional strain changes that occur as the genome is remodelled in spermatids. Conversely, DSBs associated with G-quadruplexes are more prominent in enterocytes. This is expected since G-quadruplexes lead to DSBs due to stalled replication when the replisome cannot unwind the quadruplex. Spermatids are post-replicative cells, likely explaining why we do not observe a positive association with G-quadruplexes in this cell type (Table 1). This may also explain the much larger number of DSB locations identified in enterocytes compared to spermatids. We considered whether our results might be confounded by technical differences between DBrIC (used in the spermatid study) and sBLISS (used in the enterocyte study). The primary technical difference between the protocols is that DBrIC is carried out on purified chromatin and DNA is immunoprecipitated, while sBLISS is carried out in situ on fixed nuclei with adapter ligation. Conceivably some chromatin regions may therefore be less accessible in the sBLISS enterocyte study. However, the fact that this study detected a larger number of DSB regions than the DBrIC spermatid study indicates that chromatin accessibility is unlikely to greatly compromise DSB detection by sBLISS. While the techniques also differ in resolution, we adjusted for this in our analysis by extending the sBLISS location to match the average DBrIC peak width. Nevertheless, it remains possible that aspects of the differences between the cell types are driven by methodological differences between DSB detection methods—future work in this area could aim to profile DSB locations in a range of tissues utilising a common methodology to allow systematic exploration of this question.

Following this, we carried out a broad examination of data for 16 histone marks in spermatids in relation to the spermatid DBrIC DSB locations. Finally, we related this to downstream events including histone retention and oxidative damage in mature sperm, and a transcription factor known to bind fragile chromatin in the early embryo. We show that many of these events are strongly associated with each other. These associations in turn explain aspects of the overall chromosomal distribution of both spermatids DSBs and these various chromatin components. Chr11 has the highest coverage of mESC ZSCAN4, predicted Z-DNA (positively associated with spermatid DSB locations) and experimental G-quadruplexes (negatively associated with spermatid DSB locations). Conversely chrY has the lowest coverage of mESC ZSCAN4, predicted Z-DNA and experimental G-quadruplexes. Taken together, our results suggest the presence of a common ‘vulnerability code’ that predisposes specific regions of the paternal genome to damage at several stages of the reproductive cycle. The reasons for these associations remain to be established experimentally. However, the association between spermatid DSB locations and predicted Z-DNA regions is most likely related to the torsional changes discussed above.

Torsional strain can also lead to non-B DNA formation and in particular to the formation of Z-DNA due to unwinding stress, such as that generated during protamination. Kim *et al.* (46) have shown that TG repeats preferentially formed Z-DNA over CG repeats, as the free energy barrier of the transition from B to Z-DNA was lower for TG repeats than CG repeats. They also showed that more torsional stress is required for the formation of Z-DNA in TG repeats than that required in CG repeats, as Z-DNA in TG repeats is less stable. The lower free energy barrier to form Z-DNA in TG/CA repeats might account for the enrichment of this specific motif that we observe in the spermatid DSB locations. We therefore hypothesise that B-DNA to Z-DNA transitions in specific regions of the genome may act as a molecular ‘crumple zone’, either buffering the torsional strain until it can be relieved by strand scission and helix unwinding, or simply acting in concert with topoisomerase activity to relieve the accumulated tension. While DSB locations in spermatids do not associate with the canonical topoisomerase II motif, it may be that conversion to Z-DNA facilitates topoisomerase cleavage and/or modulates its binding site specificity. Work with Drosophila topoisomerase II (47,48) showed that topoisomerase II can bind and cleave Z-DNA and it has a higher affinity for Z-DNA than B-DNA. Choi *et al.* (49) suggested that both Z-DNA and B-DNA appear to be equally attractive as topoisomerase II cleavage sites, however a supercoiled substrate (such as Z-DNA) enhanced the cleavage efficiency of topoisomerase II without altering the specificity. Work by Szlachta *et al.* (10) has shown that in human cell lines regions of the genome that have a higher potential to form stable DNA secondary structures are more prone to DSBs induced by topoisomerase II compared to random and flanking sequences.

Collectively, this work helps to explain the positive association we have observed with spermatid DSB locations and predicted Z-DNA, in that topoisomerase may preferentially cleave DNA in the Z-form giving the positive association with spermatid DSB locations. Alternatively, non-B DNA structures might lead to DNA damage through a topoisomerase-independent mechanism. The optimal substrates for the mismatch repair (MMR) proteins share similar features with some non-B DNA structures, such as the junction of B- to Z-DNA (50). The junctions could be mistaken for regions of damage and an incomplete mis match repair could result in a DSB at these regions. Future experimental work will therefore be required to resolve precisely where and when Z-DNA forms during sperm condensation, and how this relates to topoisomerase activity.

Following our investigation of the factors associated with DSBs during spermatid development, we turned our attention to potential downstream consequences of this damage. We show here for the first time that retention of histones in mature sperm is positively associated with DSB locations arising several days previously, during spermatid elongation (Figure 2C). This implies that DSB formation during chromatin condensation selectively impairs local replacement of histones with protamines. This could be a direct effect, if the presence of DNA repair factors prevents access by the protamination machinery. Alternatively, it may be an indirect effect mediated by Z-DNA, if Z-DNA is refractory to removal of histones and replacement by protamines (51) and thus protamination in one region of the genome could trigger refolding of nearby ‘crumple zones’ into Z-DNA and prevent them in turn becoming protaminated. Despite the association between spermatid DSB locations and retained histones in sperm, we found that DSBs were negatively associated with many histone modifications in spermatids, with the only positive associations being with lysine acetylation (a core event during histone eviction) and components of the spermatid DNA damage response (BRD4, H3K9me3 and H2AZ). It is possible that high coverage of a chromosome with histone marks may reduce its susceptibility to damage, for example chromosome 11 had the highest coverage of states E1 and E2 (Figure 3) and it has the lowest coverage of DSBs. Conversely the Y chromosome has the second lowest coverage of state E1 and the lowest coverage of state E2 and it has the highest coverage of DSBs. Alternatively, histone proteins may be removed at DSB sites to facilitate the repair process (52).

We also showed that regions of high oxidative damage are also positively associated with spermatid post-meiotic DSB locations, predicted Z-DNA, STRs and retained histones in sperm. It remains to be established whether spermatid DSB locations intrinsically prime sperm chromatin for oxidative damage, or whether the effect is mediated indirectly via histone retention, since the DNA of regions retaining histones in mature sperm is less compact and therefore more susceptible to OD. Intriguingly, other work shows that the guanines in Z-DNA are more sensitive to alkylating modifications than in B-DNA (53,54). Once these modifications have formed on Z-DNA they are resistant to excision by repair enzymes (55). If this applies also to oxidative damage it could provide an alternative mechanism for the co-localisation of sperm oxidative damage with spermatid DSB locations and Z-DNA. There are clearly multiple factors that predispose sperm chromatin to OD since we (and others (56)) also observe a correlation between OD and G-quadruplex regions. It is unclear why G-quadruplexes should be susceptible to OD in mature sperm as these are neither associated with spermatid damage nor histone retention. It may be that this is an artefactual association since OD in this study was defined as the presence of the oxidized base 8-hydroxy-2′-deoxyguanosine (8OHdG), which will thus inevitably be more prevalent in G-rich regions capable of G-quadruplex formation. Susceptibility of the chromosomes to external oxidative damage has been reported to depend upon their position within the sperm head, with a peripheral or basal location being more susceptible to oxidative attack (57).

Finally, we showed that spermatid DSB locations (and other associated features such as predicted Z-DNA and CA repeats) were also strongly associated with genomic regions that bind ZSCAN4 in embryonic stem cells with two-cell like features. Importantly, the association between spermatid DSB locations and two-cell like ZSCAN4 was even stronger when we focused on DSB locations occurring outside CA repeats, indicating that this is not driven solely by the proposed CA binding activity of ZSCAN4. Rather, it may be that ZSCAN4 is recognising Z-DNA directly. Regardless of the precise mechanisms, our results demonstrate a continuity of genomic localisation of damage-associated factors from spermatid elongation, through the mature sperm, up to embryonic development. As the earliest event in this chain, it seems possible that chromatin remodelling events in spermatids not only trigger DSBs during chromatin condensation, but may also precondition DNA for damage from multiple sources later on in reproduction.

The results presented here are the associations between different marks in spermatids, retained histones in epididymal sperm and predicted non-B DNA. Without single cell data we cannot unequivocally determine whether a DSB occurs in the exact region of a particular histone modification. Even with single cell data, it is impossible to measure the same cell at different stages of its life history without the development of non-destructive techniques for chromatin profiling. Thus, while we show that spermatid DSB locations are associated both with predicted Z-DNA and subsequently with regions retaining histones in epididymal sperm, we cannot say for sure whether histone retention is a consequence of changes in DNA conformation, or simply occurs in the same genomic regions. We note however that DSBs themselves are rare events affecting only a small fraction of cells and thus the downstream events in mature sperm and the early embryo are not direct consequences of DSBs occurring in spermatids. There may be different sequences of events leading to the associations that we have observed. One possibility is that histone retention at specific fragile sites is necessary to retain them in the B-DNA form. In the rare cells where histones are removed from these sites, this triggers refolding into Z-DNA and vulnerability to strand breakage. Under this hypothesis, the signals dictating histone retention at these sites remain to be elucidated. Alternatively, it may be that these sites constitutively fold into Z-DNA during spermiogenesis, and that this directly prevents histone replacement and leads to histone retention. Under this alternative hypothesis, the triggers for refolding into Z-DNA and the kinetics for re-establishment of B-DNA following fertilisation remain to be determined. Direct profiling of Z-DNA at different stages of spermiogenesis may in time resolve some of these questions.

**Figure 4.**
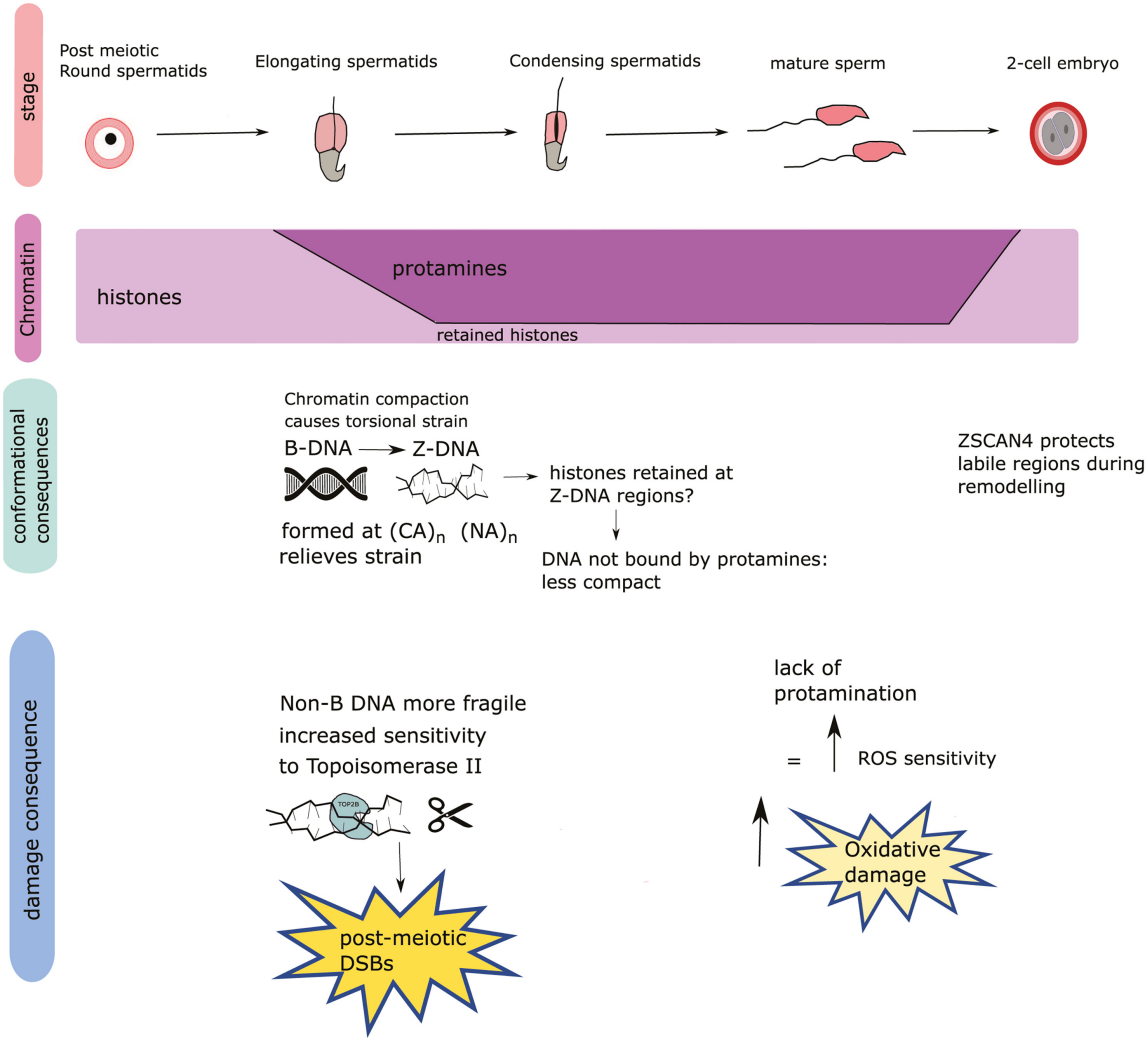
Summary hypothesis schematic of spermiogenesis and the possible role of Z-DNA in DSB formation. The stage track shows different stages of spermiogenesis. The chromatin track shows the changes that occur in chromatin as spermiogenesis progresses. The conformational consequences track shows the progression of chromatin changes through reproduction, while the damage consequence track shows the consequences for different types of DNA damage at each stage. We propose that DSBs in spermatids effectively act as a ‘tracer’ for regions undergoing remodelling due to torsional changes, while oxidative damage in mature sperm ‘traces’ regions that are vulnerable due to histone retention. These regions are substantially shared, because some aspect of the remodelling process triggers histone retention and thus ongoing vulnerability to damage.

In summary, we have shown that spermatid DSB locations are positively associated with specific primary and secondary DNA structures, and with a limited range of histone modifications. These same genomic regions are then associated with damage and damage-control factors both in the mature sperm and the embryo, signifying the presence of a common ‘vulnerability code’ for the paternal genome throughout reproduction. We conjecture that the switch from B to Z-DNA acts as a molecular crumple zone to help relieve the torsional strain that occurs during chromatin remodelling in spermatids, with the caveat that the Z-DNA is more prone to DSBs than B-DNA possibly by preferential topoisomerase II cleavage (Figure 4). Some aspect of the remodelling process—potentially DNA conformational change, or alternatively the repeated severing and re-joining of DNA by topoisomerases—subsequently has impacts on protamination and histone retention in sperm, in turn affecting oxidative damage. Finally, these same regions once again become vulnerable as protamines are replaced by histones in the embryo. Understanding how all classes of non-B DNA and epigenetic marks integrate with the NHEJ pathway and the DNA damage response will further our understanding of DNA damage and paternal genome mutagenesis throughout the reproductive cycle.

## DATA AVAILABILITY

No new data was generated for this study. Code used for plotting can be found at https://github.com/Farre-lab/Spermatid_DSB_paper and https://zenodo.org/record/7433522#.Y5iePXbP1PZ.

## ACCESSION NUMBERS

All data files used for this analysis are publicly available and can be downloaded from the NCBI GEO repository. Accession numbers for the files are:

DSB data: ERX1946916, ERX1946917, ERX1946918, input: GSM1046836

Oxidative damage data: ERX1236388, ERX1236389, ERX1236390

Spermatid histone data: H3K4me3 GSM1046840, GSM1046841 and GSM1202707, H3K27me3 GSM1046842, GSM1046843 and GSM1202710, H3K4me1 GSM1202712, H3K27ac GSM1202715, input GSM1202725, 5hmC GSM1202718, GSM1202722 H2AZ, Kac GSM810677, Kcr GSM810678, BRD4 GSM1519002, H3K9me3 GSM1519003, H3K9ac GSM1519004, H4K5ac GSM1519005, H4K8ac GSM1519006, H4K12ac GSM1519007, H4K16ac GSM1519008 and H4Kac GSM1519009.

Retained histones DRX117176 and DRX117177

Enterocyte sBLISS: GSM4322063 and GSM4322066

Experimental G-quadruplex: GSM3003548

ZSCAN4: GSM4175885

Predicted Z-DNA/STR and G-quadruplex downloaded from the non-B DB for mouse mm10.

## Supplementary Material

gkad089_Supplemental_Files

## Notes

Present address: Peter Ellis, Marta Farré, School of Bioscience, University of Kent, Canterbury CT2 7NJ, UK.
