# Supplementary material for: A shared ‘vulnerability code’ underpins varying sources of DNA damage throughout paternal germline transmission in mouse
Source: Nucleic Acids Res. 2023 Feb 20;51(5):2319–32. doi: 10.1093/nar/gkad089 (PMC10018361; doi:10.1093/nar/gkad089)
Supplement: gkad089_Supplemental_Files [file gkad089_supplemental_files.zip › Burden_et_al_all_suppl_Fig_edited_upsetR_new_fig6_R2.pdf]

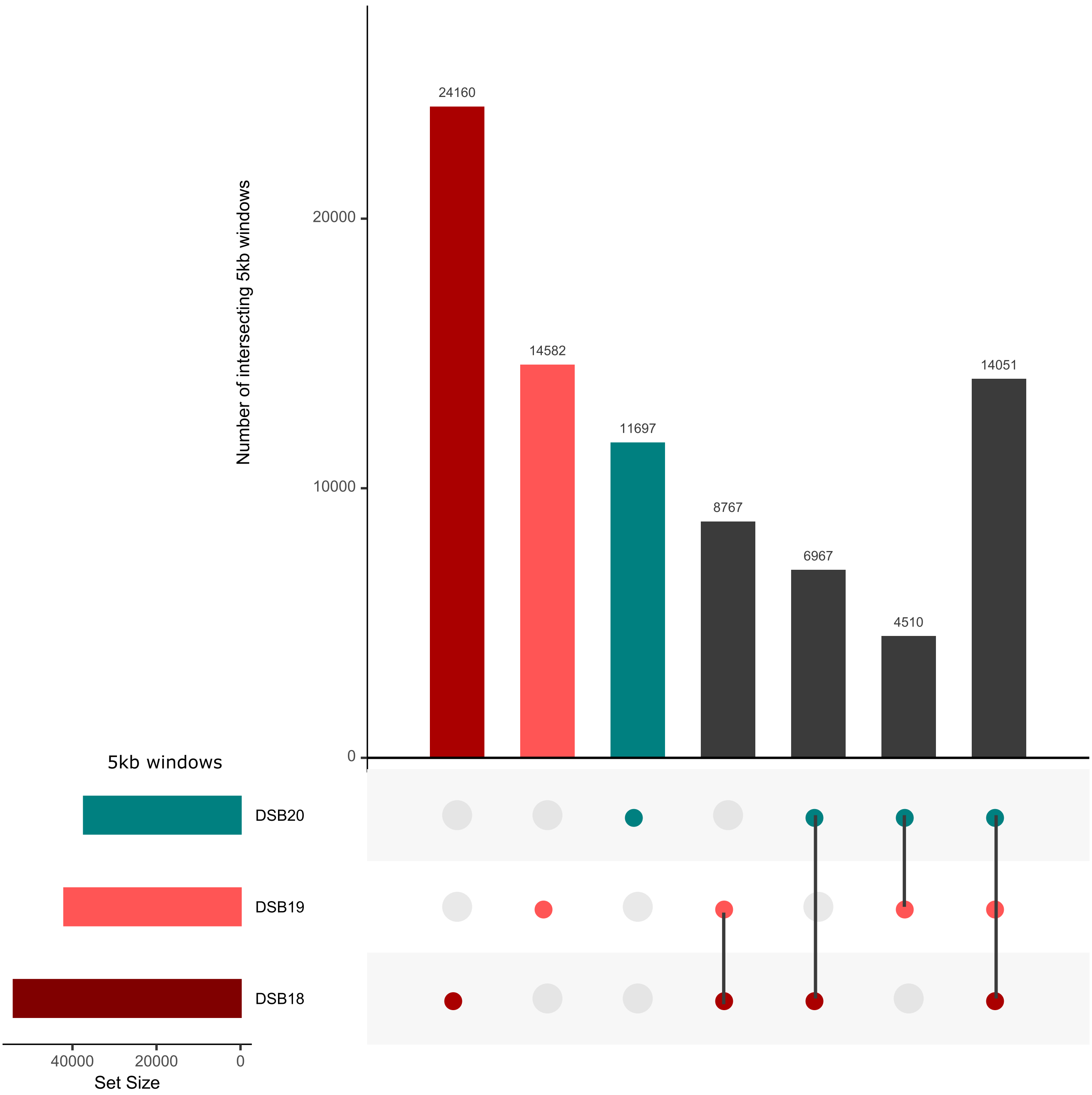

Supplementary Figure 1: UpSetR plot showing the overlapping and unique 5kb windows between the round spermatid DSB files 18/19 and the condensing spermatid file DSB20, related to Figure 1A. The number of 5kb windows with a DSB signal in each file is represented on the left barplot as 'set size' The X-axis represents the number of 5kb windows containing a DSB signal for the different overlap combinations. Different combinations of overlap are represented by the black lines interlinking the coloured circles. The DSB18/19 files are round spermatids stages 1-9 (shown in red and pink bars) and represent the total number of 5kb windows containing a signal unique to these files. The DSB20 file (shown in the blue bar) is condensing spermatids stage 15-16 and this peak also represents the total number of 5kb windows containing a DSB unique to this file. Bars in dark grey represent the number of 5kb windows with signal in more than one file.

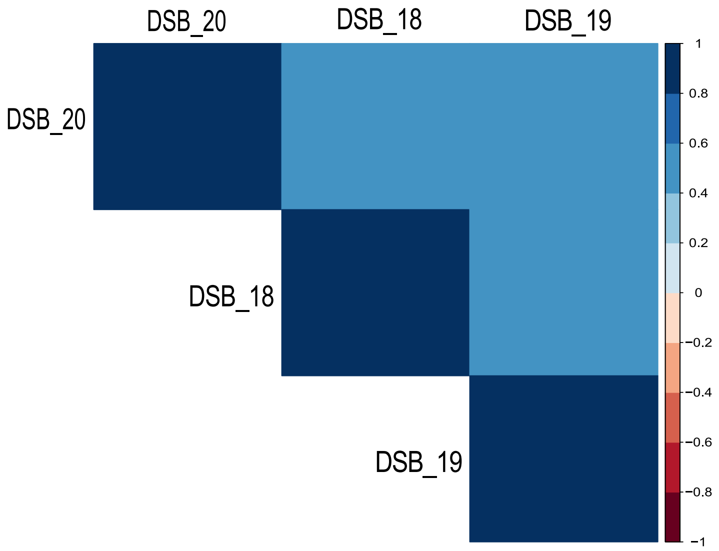

Supplementary Figure 2: Correlation of the post-meiotic spermatid DSB files using 1kb windows, related to Figure 1A and supplementary Figure 1. DSB18/19= round spermatids and DSB20=condensing spermatids

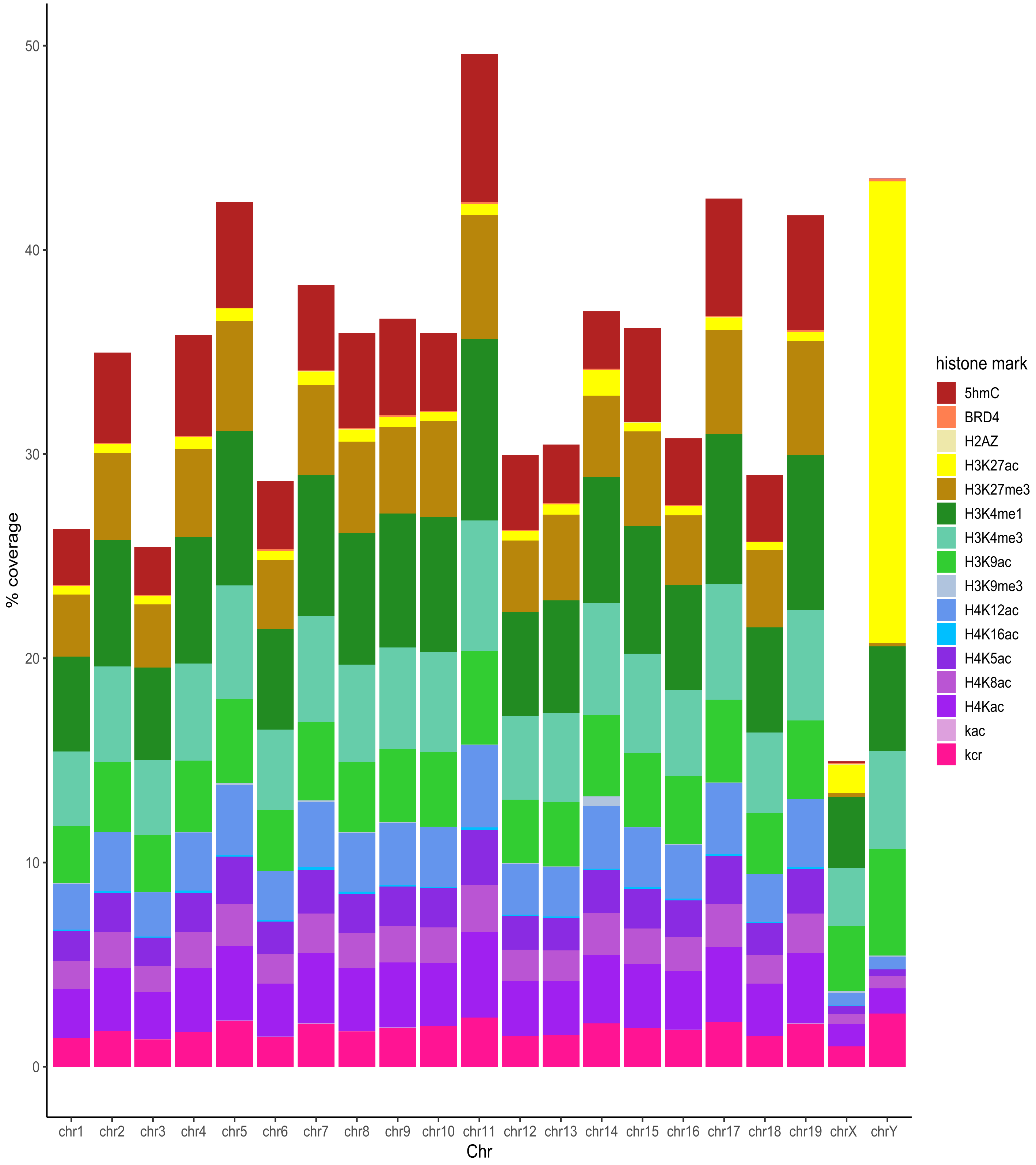

Supplementary Figure 3: Coverage of the 16 histone marks used for ChromHMM analysis in Figure 3, related to Figure 3A.

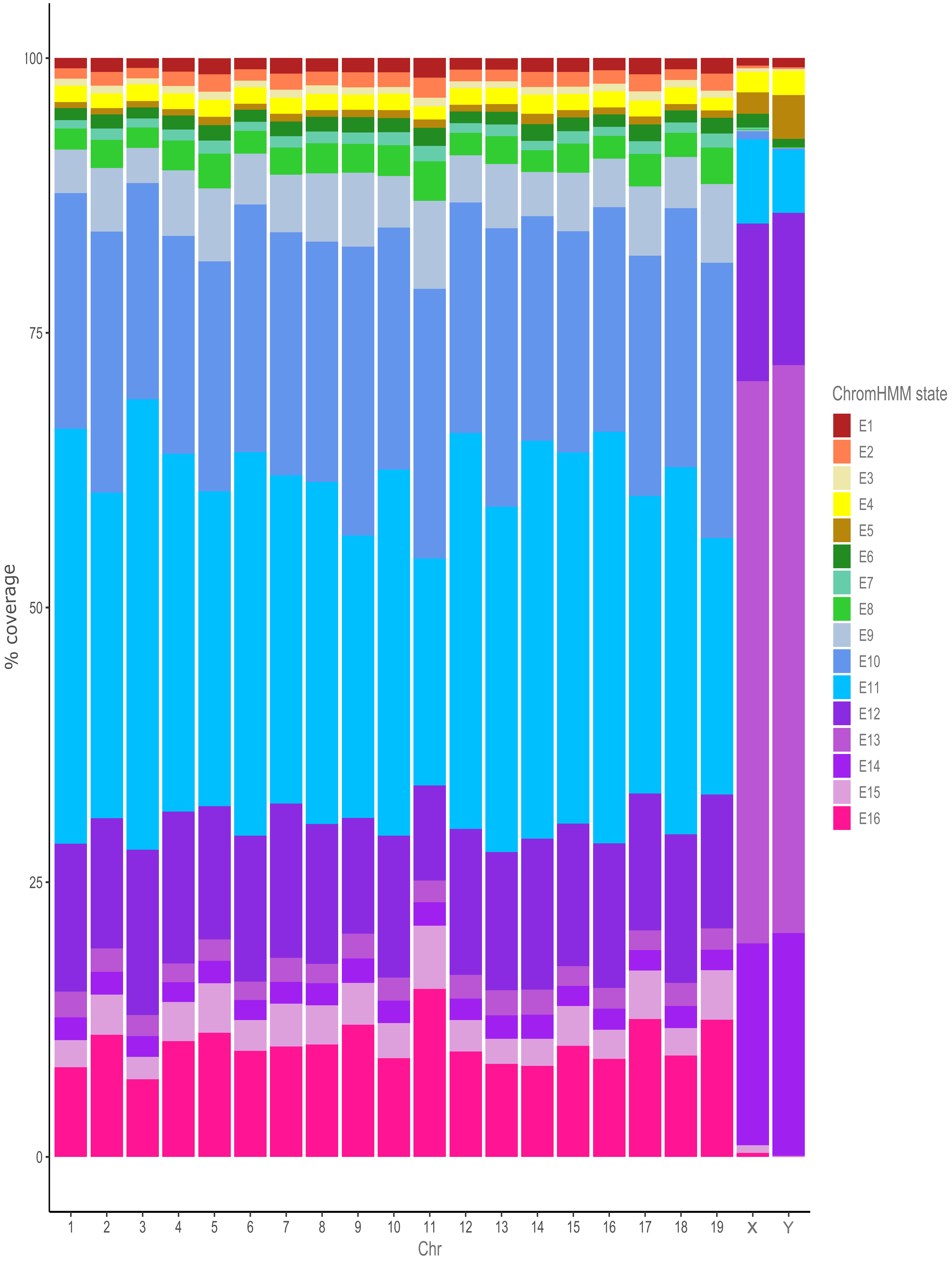

Supplementary Figure 4: Coverage of the 16 spermatid chromHMM states, related to Figure 3A.

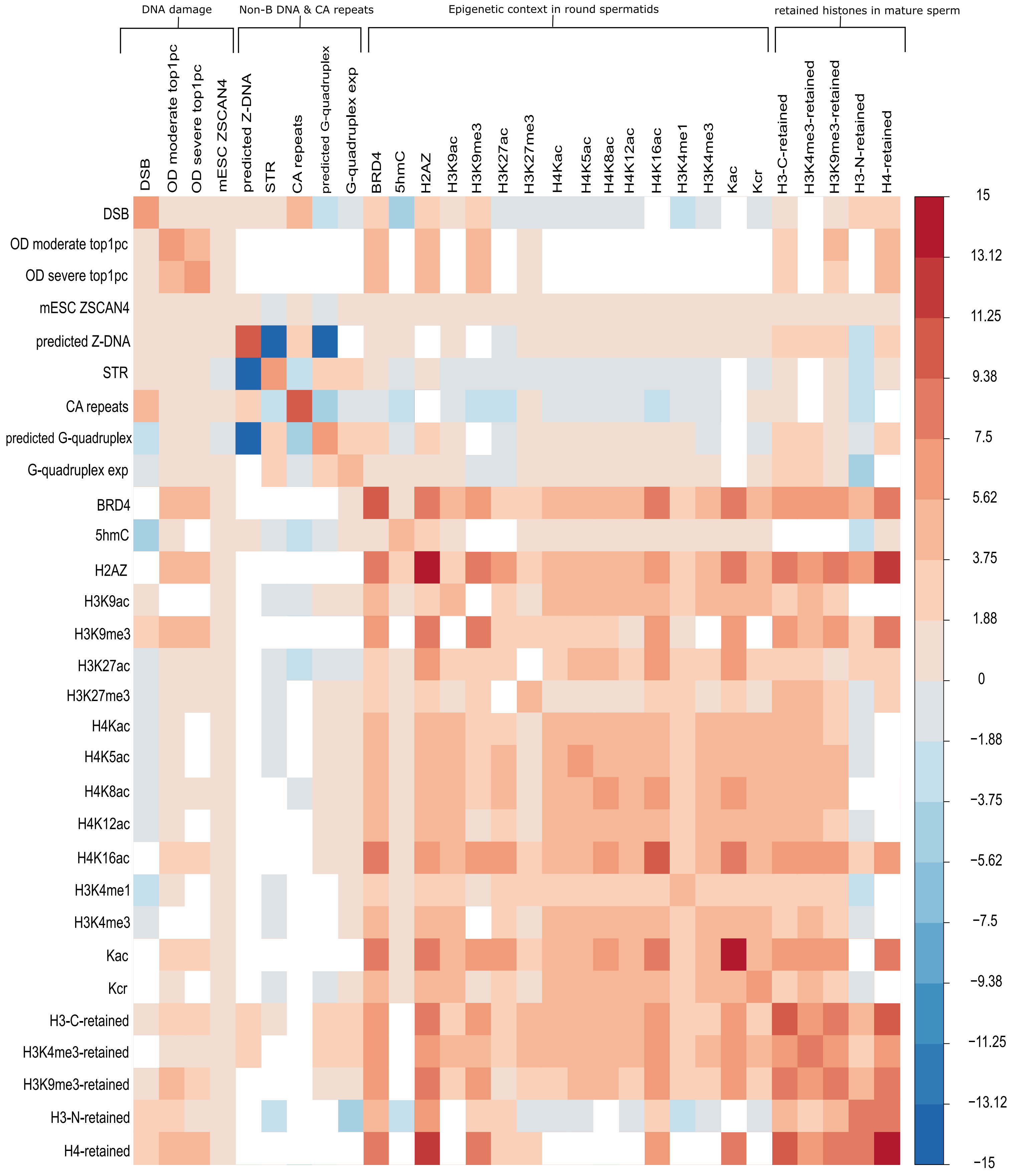

Supplementary Figure 5: Permutation association of DNA damage, non-B DNA repeats, epigenetic context in round spermatids and retained histones in mature sperm. Genomic association tester (GAT) heatmap showing the log2 fold change between samples. Shades of red show significant positive log2 fold change & shades of blue show significant negative log2 fold change. Non-significant associations with a P-value of > 0.01 are shown in white. In cases where fold change was 0, the log2 fold change has been fixed on this plot as –14.

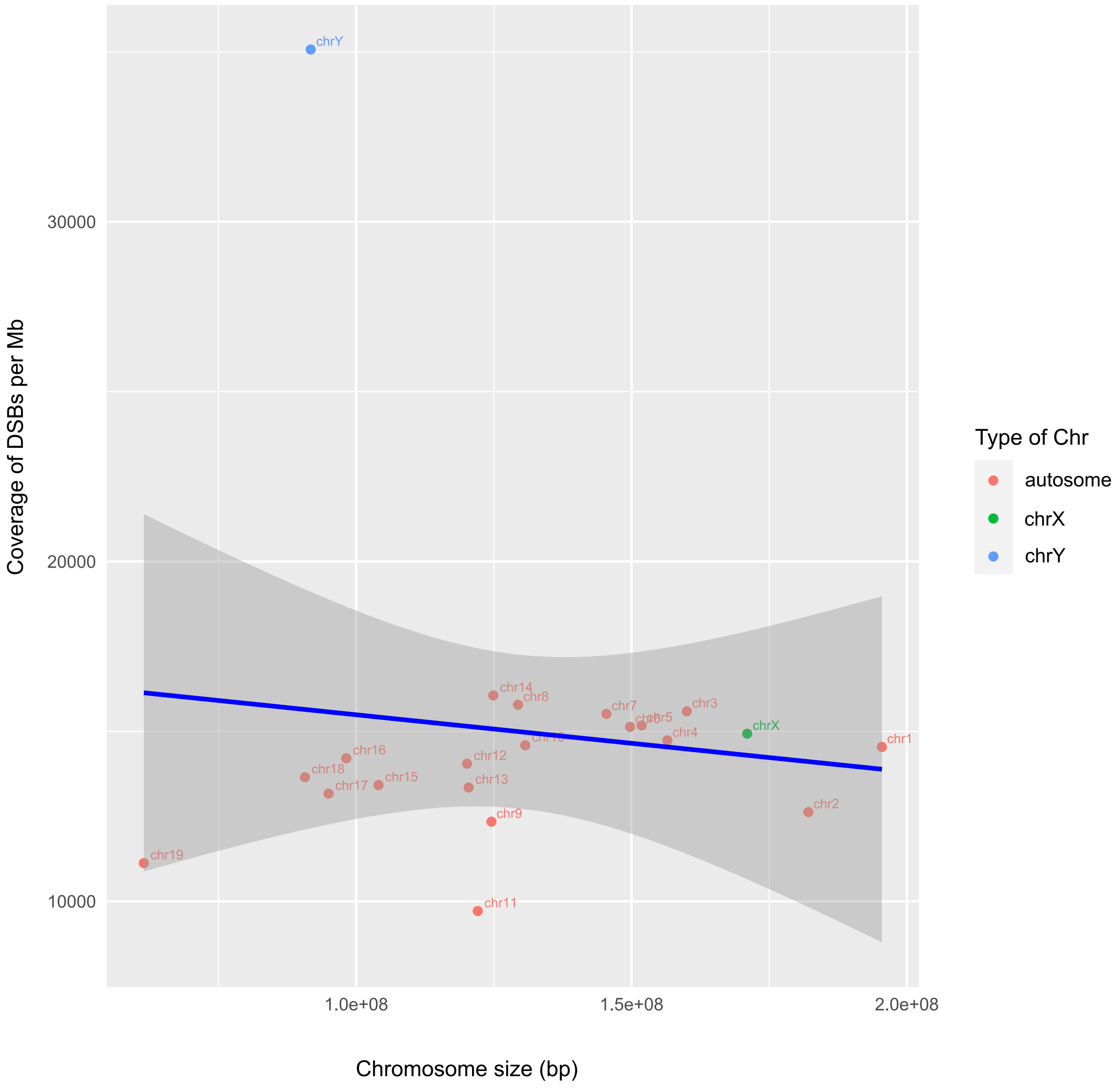

Supplementary Figure 6: Correlation between post-meiotic DSBs and chromosomal size. Linear regression of the number of post-meiotic DSBs (expressed as total bp coverage per Mb) detected in mouse chromosomes. Autosomes are depicted in red, the X chromosome in green and the Y chromosome in blue. Grey shading represents 95% confidence interval.

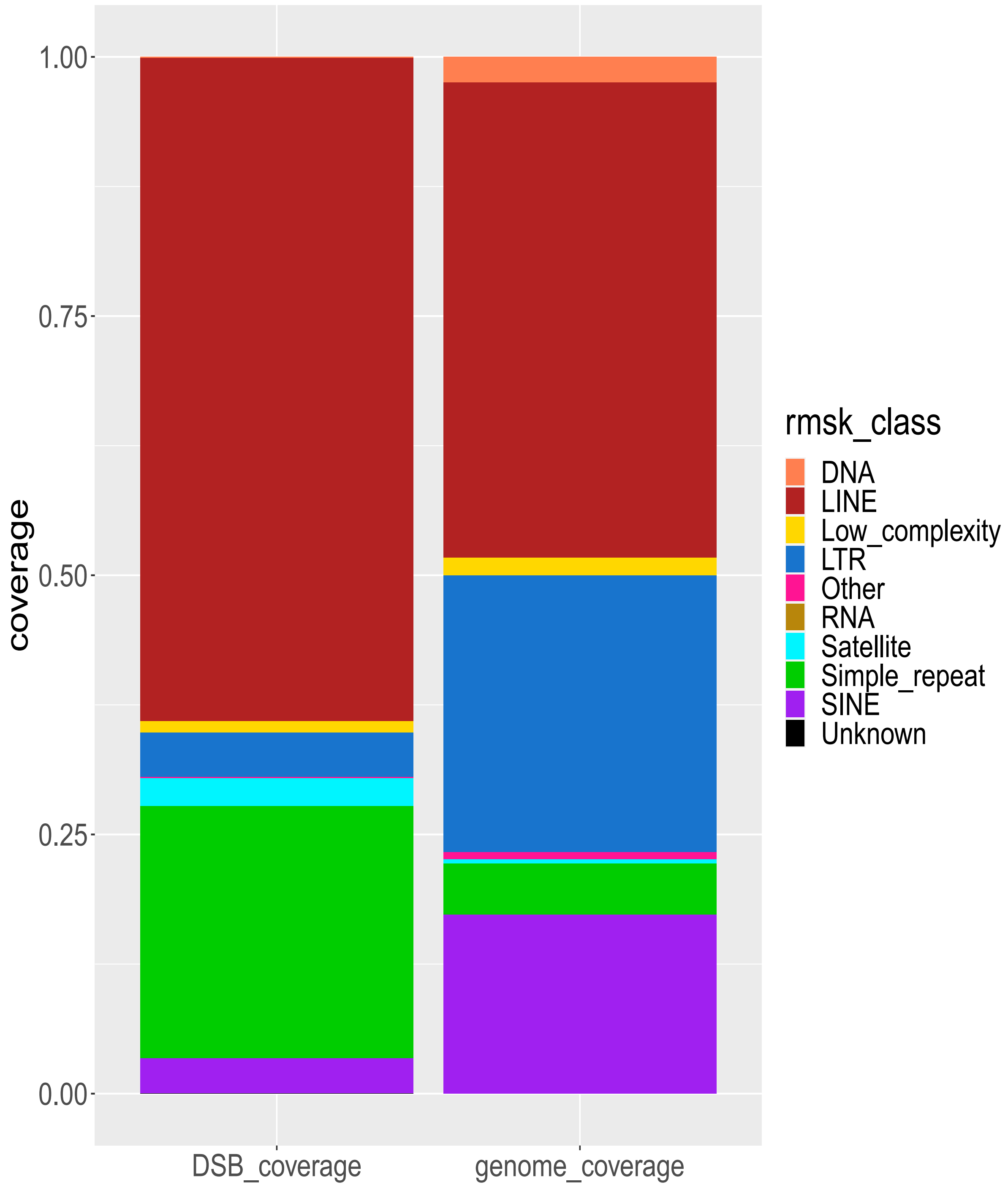

Supplementary Figure 7: Coverage of transposable elements within the post-meiotic mouse spermatid DSBs vs the whole genome.
